# The function of metformin in endometrial receptivity (ER) of patients with polycyclic ovary syndrome (PCOS): a systematic review and meta-analysis

**DOI:** 10.1186/s12958-021-00772-7

**Published:** 2021-06-14

**Authors:** Lifang Yuan, Hongbo Wu, Weiyu Huang, Yin Bi, Aiping Qin, Yihua Yang

**Affiliations:** 1 Reproductive Medical Center, the First Affiliated Hospital of Guangxi Medical University, Nanning, China; 2 Reproductive Medical Center, Qinzhou Women and Children Hospital, Qinzhou, China

**Keywords:** Metformin, PCOS, Endometrial receptivity, Endometrial thickness, Meta-analysis

## Abstract

**Background:**

This meta-analysis summarizes evidence from studies using metformin (Met) to improve endometrial receptivity (ER) in women with PCOS.

**Methods:**

Following the PRISMA protocol, we conducted a comprehensive search of academic literature from various databases, including PubMed, EMbase and Cochrane libraries. Studies published in English before Jan 27, 2021, were recruited for primary screening. Data on endometrial thickness (EMT), endometrial artery resistance index (RI), clinical pregnancy rate (CPR) and miscarriage rate (MR) were extracted and analyzed.

**Results:**

Sixty-two eligible studies that included 6571 patients were evaluated in this meta-analysis. Primary indicators are EMT and endometrial aetery RI; secondary indicators include the clinical pregnancy rate and miscarriage rate. Metformin significantly increased EMT (SMD = 2.04, 95% CI (0.96,3.12),*P* = 0.0002) and reduced endometrial artery RI compared to the non-Met group (SMD = − 2.83, 95% CI: (− 5.06, − 0.59), *P* = 0.01). As expected, metformin also improved CPR and reduced MR in PCOS patients as a result, clinical pregnancy rate (risk ratio [RR] = 1.26, 95% CI: 1.11–1.43, *P = 0.*0003), and miscarriage rate (RR = 0.73, 95% CI:0.58–0.91, *P* = 0.006).

**Conclusion:**

Metformin may improve endometrial receptivity (ER) in PCOS patients by increasing EMT and reducing endometrial artery RI. However, the level of most original studies was low, with small sample sizes. More large-scale, long-term RCTs with rigorous methodologies are needed.

## Introduction

During recent decades, assisted reproductive technologies (ARTs), such as IUI, in vitro fertilization (IVF) and embryo transfer (ET), have become increasingly popular. Move over, the prognosis of ART treatment for infertility patients has greatly improved; however, some patients still cannot achieve clinical pregnancy after receiving multiple high-quality embryo transfers. There are many causes that may explain such implantation failure, and endometrial receptivity (ER) is critical [1]. By definition, ER refers to the ability of the endometrium to allow the blastula to locate, adhere, invade, and ultimately implant during the period when the endometrium matures (luteal phase) [2]. A successful pregnancy requires synchronization of the development of the embryo and a receptive endometrium [3]. It is generally accepted that the quality of the embryo and ER play an important role in pregnancy establishment and maintenance. However, improving ER remains a challenge for clinicians [4], and it is an crucial strategy to improve the live birth rate [5].

As an insulin sensitizer, metformin has been widely used in infertility clinics. It is generally applied to reduce insulin resistance and glucose metabolism abnormalities in PCOS patients [6]. To improve the pregnancy rate of PCOS patients, metformin has been widely used alone or in combination with clomiphene for ovulation induction [7], though the impact of ER on PCOS patients is still controversial.

Recently, many meta-analyses and systematic reviews have been conducted on women with infertility and PCOS treated with metformin, such as the ovulation rate, pregnancy rate, miscarriage rate, serum sex hormone levels, and adverse effects [8]. Nonetheless, few meta-analyses and systematic reviews have addressed the effects of metformin on ER in PCOS patients. The main purpose of this study was a comprehensive systematic review and meta-analysis to compare the effects of metformin used alone or in combination on ER in PCOS patients.

## Methods

### Search strategy

Strictly following the Preferred Reporting Items for Systematic Reviews and Meta-analyses (PRISMA) 2009 Checklist Protocol, two independent reviewers (Y.B. and W.Y.H) performed a literature search in three electronic databases (PubMed, EMbase, and Cochrane Library). Studies published before May 31, 2020, were retrieved using the following key words: “Metformin” [Mesh] OR Dimethylbiguanidine OR Dimethylguanylguanidine OR Glucophage OR Metformin Hydrochloride OR Hydrochloride, Metformin OR Metformin HCl OR HCl, Metformin AND (“polycystic ovary syndrome” [Mesh] OR Ovary Syndrome, Polycystic OR Syndrome, Polycystic Ovary OR Stein-Leventhal Syndrome OR Stein Leventhal Syndrome OR Syndrome, Stein-Leventhal OR Sclerocystic Ovarian Degeneration OR Ovarian Degeneration, Sclerocystic OR Sclerocystic Ovary Syndrome OR Polycystic Ovarian Syndrome OR Ovarian Syndrome, Polycystic OR Polycystic Ovary Syndrome 1 OR Sclerocystic Ovaries OR Ovary, Sclerocystic OR Sclerocystic Ovary). Preselected results were limited to English publications. Manual searches were also conducted to acquire potentially eligible articles that might have been missed by computer-based searches.

### Study selection

Two investigators (W.Y.H and Y.B) reviewed the titles and abstracts of all literatures identified by the search strategy to generate a list of relevant articles, and the full texts were searched and read by another two reviewers (L.F.Y and Y.X.Z). Any disagreement was resolved by discussion or based on the judgment of a third expert (Y.H.Y) until a consensus was reached.

### Eligibility criteria

Studies were considered eligible for the meta-analysis and systematic review if they met the following inclusion criteria: (i) experimental or observational studies; (ii) subjects received treatment alone or in combination, and indicators related to ER were assessed. The exclusion criteria were as follows: (i) case reports and reviews; (ii) patients with endometrial hyperplasia, endometrial cancer, uterine myoma, endometrial polyps, intrauterine adhesion (IUA), and other pathological changes that could affect ER; (iii) studies published in non-English languages; and (iv) the full text could not access. Data were extracted from eligible studies by two reviewers using piloted screening forms in Microsoft Office Excel. Including the author, year of publication, number of participants, mean age, intervention, and time to measure EMT. The details of each outcome measure, such as EMT, endometrium artery RI, clinical pregnancy rate, and miscarriage rate, were precisely recorded.

### Statistical analysis

The meta-analysis was conducted using Cochrane Review Manager software (RevMan 5.3). Continuous outcomes were measured by Std mean differences (SMDs) and dichotomous outcomes by risk ratios (RRs), both with 95% confidence intervals (CIs). Heterogeneity measurement was performed by forest plots as well as calculating I^2^ (> 50% was considered extensive heterogeneity). A fixed-effects model was used to combine study results if heterogeneity was minimal; otherwise, the random-effects model was used. Potential publication bias was also examined qualitatively by funnel plots using RevMan software when the distribution of CI deviated significantly.

## Results

### Study inclusion and basic characteristics

**Fig. 1:**
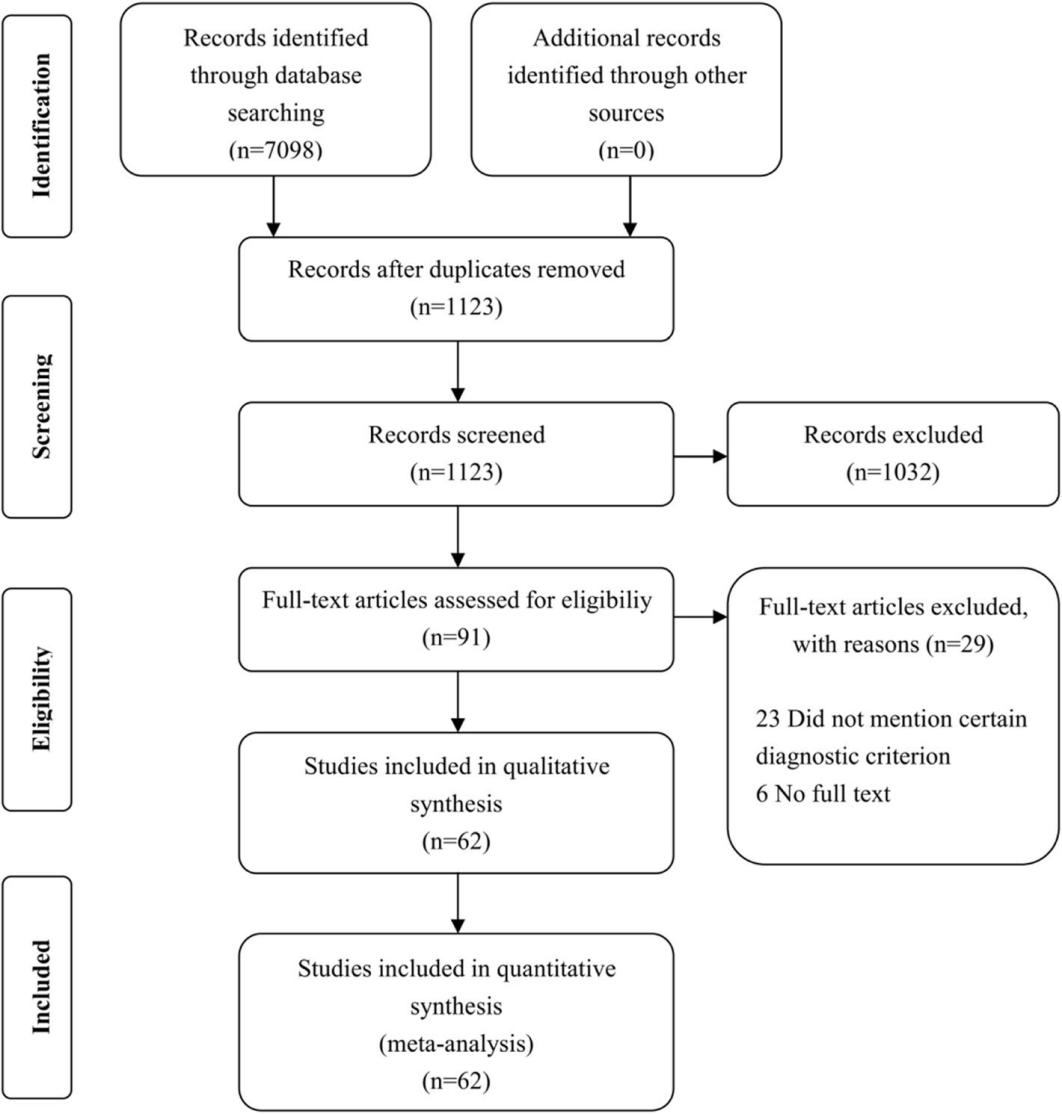
PRISMA flow diagram of study selection

The literature research initially resulted in 7098 potentially relevant publications (Medline: 1773, EMbase: 4179, Cochrane: 1146); after removing duplicates, the remaining 1123 records were screened via titles and abstracts. 91 full-text articles assessed for eligibility, after further assessment of eligibility criteria, 29 articles were excluded (23 studies did not mention certain observation indicators, and complete data were not available in the other 6 studies). Thus, a total of 62 articles were used in this study [9–70], involving 6571 patients; the studies varied from 46 RCTs, 16 cohort studies, that is, they were nonrandomized experimental studies (Fig. 1). The baseline characteristics of the included studies are presented in Table 1. The Methods and results of the meta-analysis are presented in Table 2.

**Table 1:**
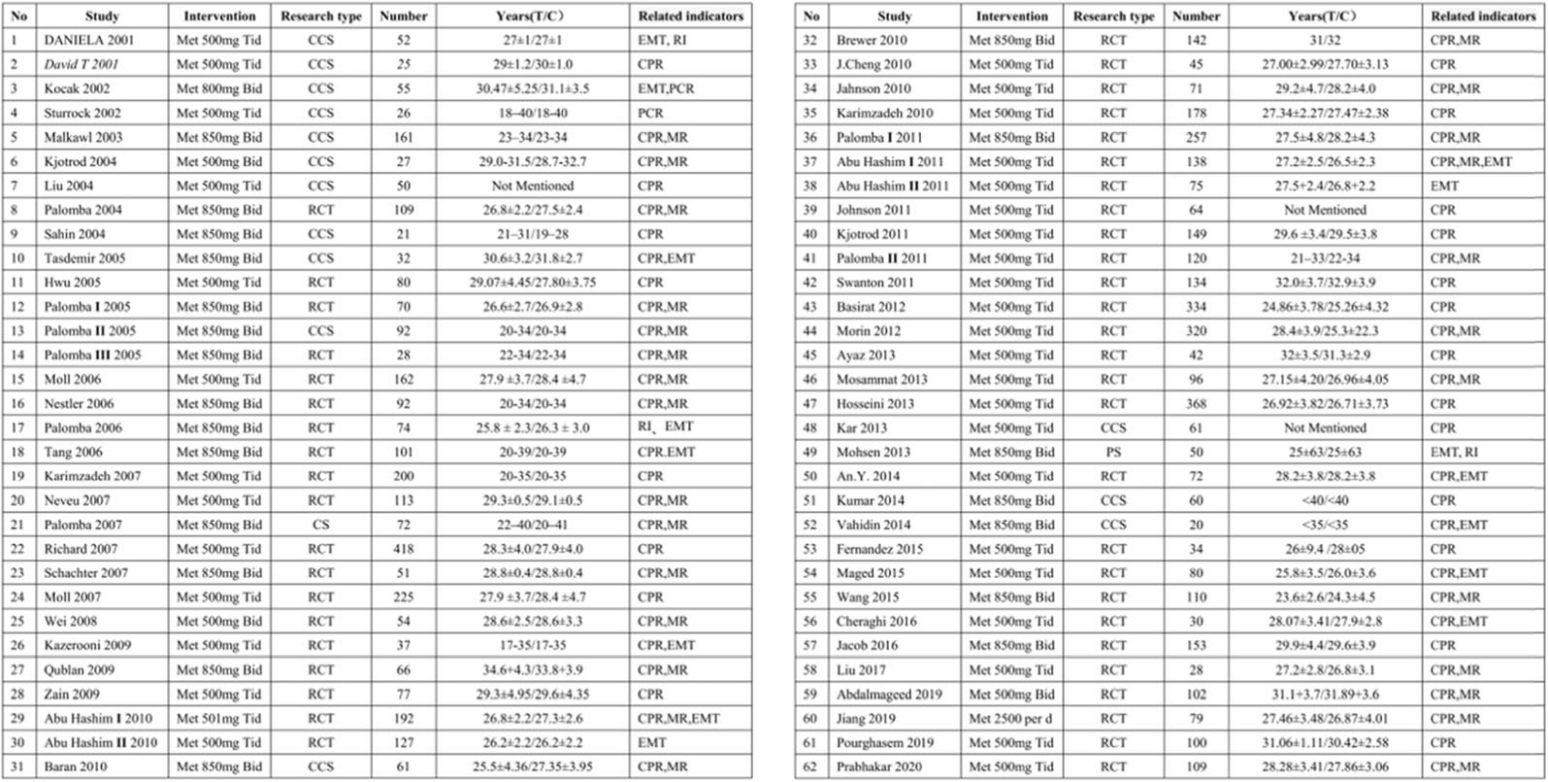
Baseline characteristics of the included studies

Abbreviation: *RCT* randomized controlled trial, *CS* cohort study, *CCS* case-control study, *PS* prospective study, *MET* metformin, *CC* clomiphene, *CPR* clinical pregnancy rate, *EMT* endo metrial thickness, *MR* miscarriage rate; *RI* resistance index, *Bid* bis in die, *Tid* ter in die

**Table 2:**
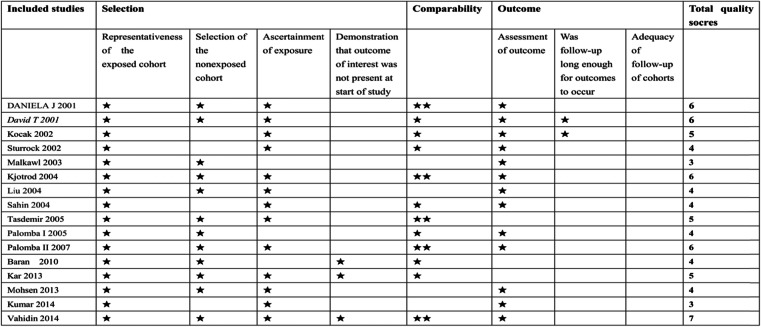
Methods and results of the meta-analysis

*CI* confidence interval, *CC* clomiphenecitrate, *LOD* laparoscopic ovarian diathermy, *L* letrozole, *MET* metformin, *M-H* mantel-haenszel, *MD* mean difference, *NAC* N-acetyl-cysteine, *OR* oddsratio, *Std* standard deviation

^**a**^Statistically significant difference

### Quality assessment of the included studies

**Fig. 2:**
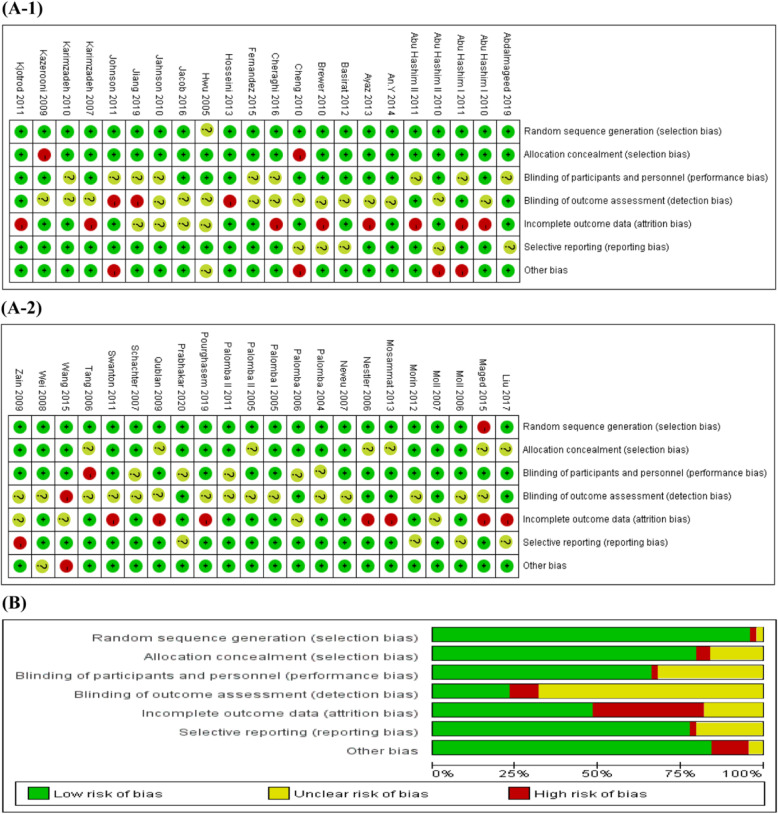
Risk of bias for eligible randomized controlled trials

Bias in the included studies was assessed by different tools. Figure 2 illustrates the risk of bias of the RCTs. Both selection and reporting bias were relatively low. The MINORS score of nonrandomized experimental studies is shown in the last column of Table 3.

**Table 3:**
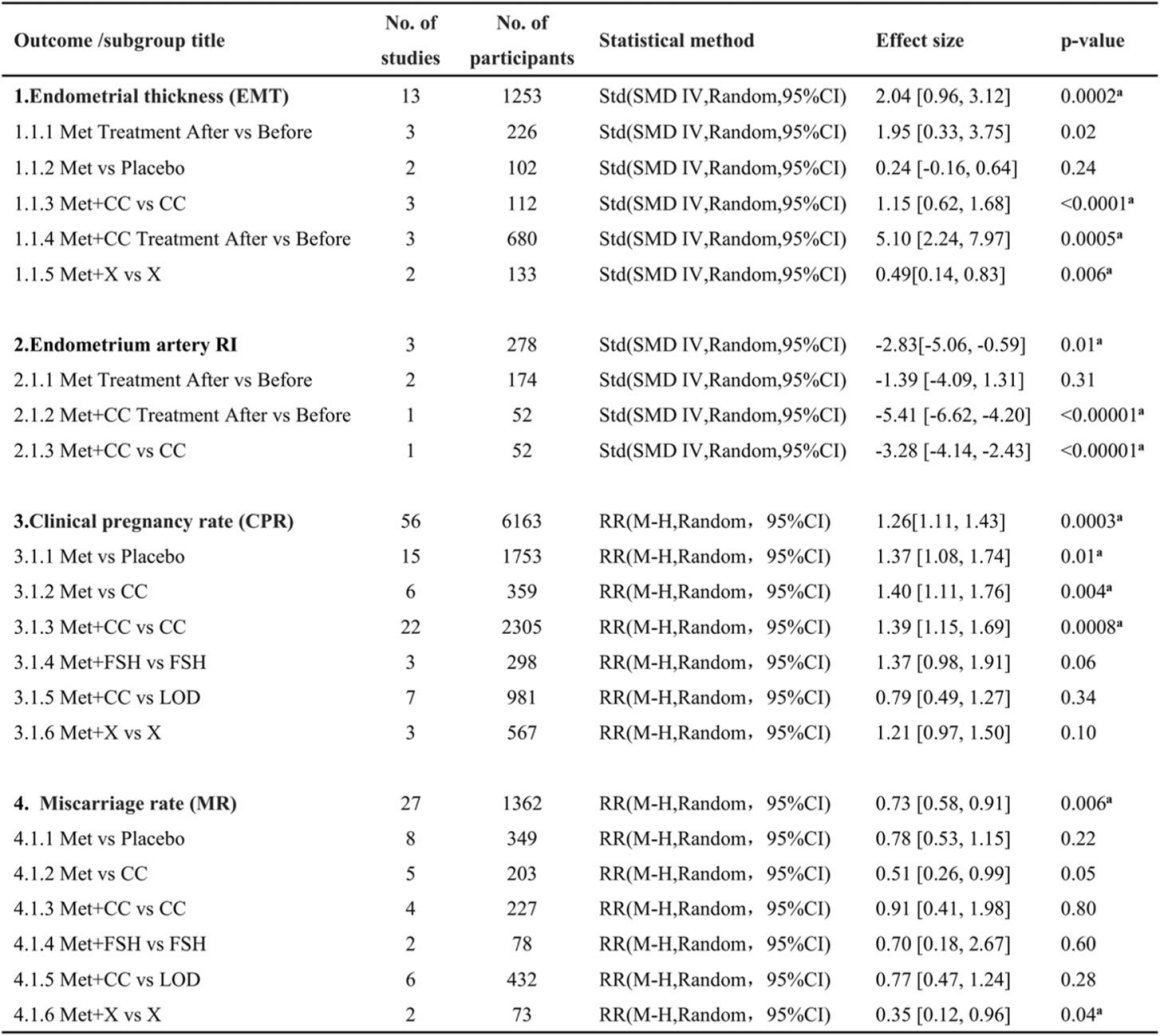
Quality assessment of the included studies

A study can be awarded a maximum of one star for each numbered item within the Selection and Outcome categories. A maximum of two stars can be given for Comparability

### Meta-analysis

**Fig. 3:**
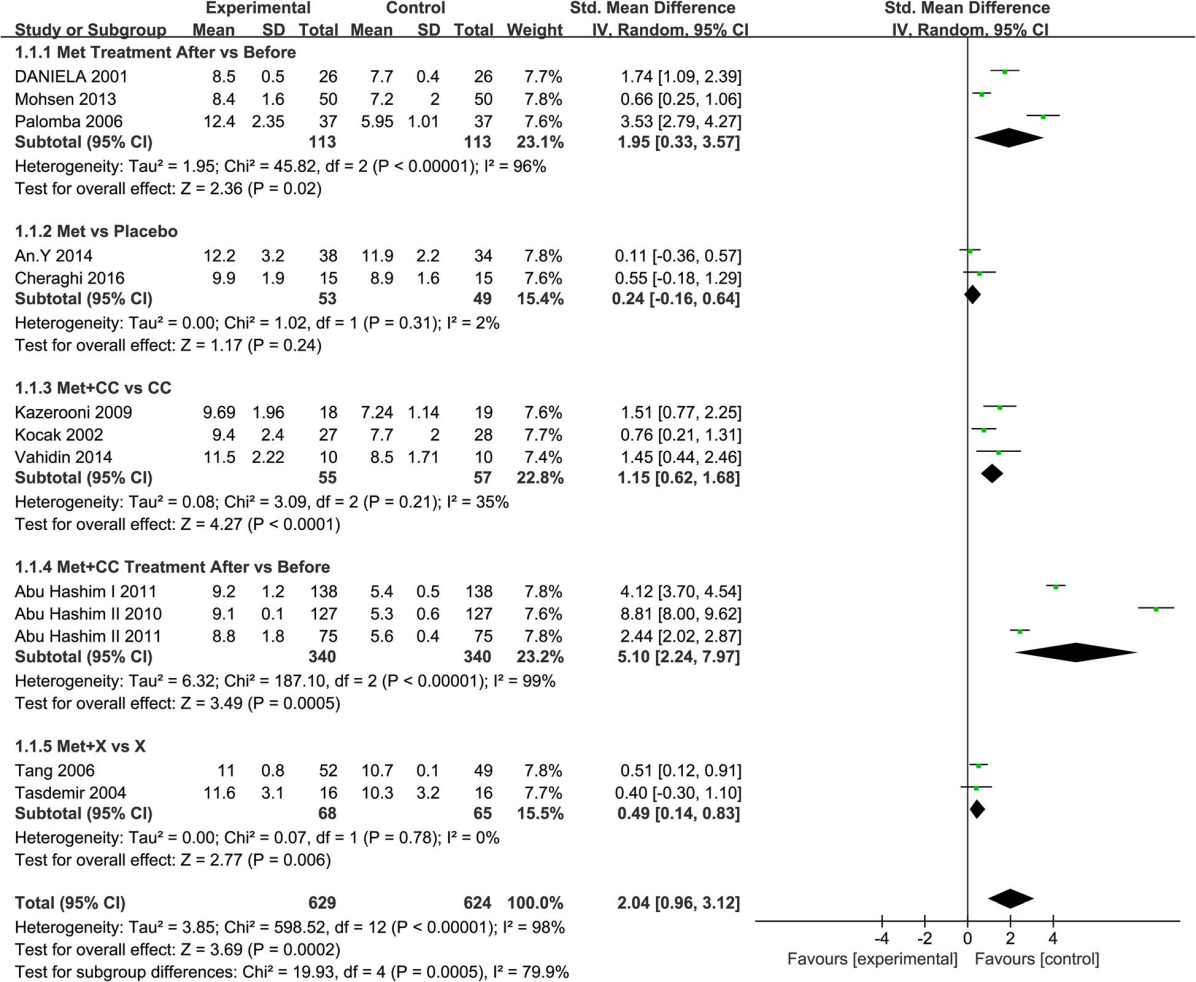
Forest plot of comparison: Met vs control, outcome: endometrial thickness (EMT)

**Fig. 4:**
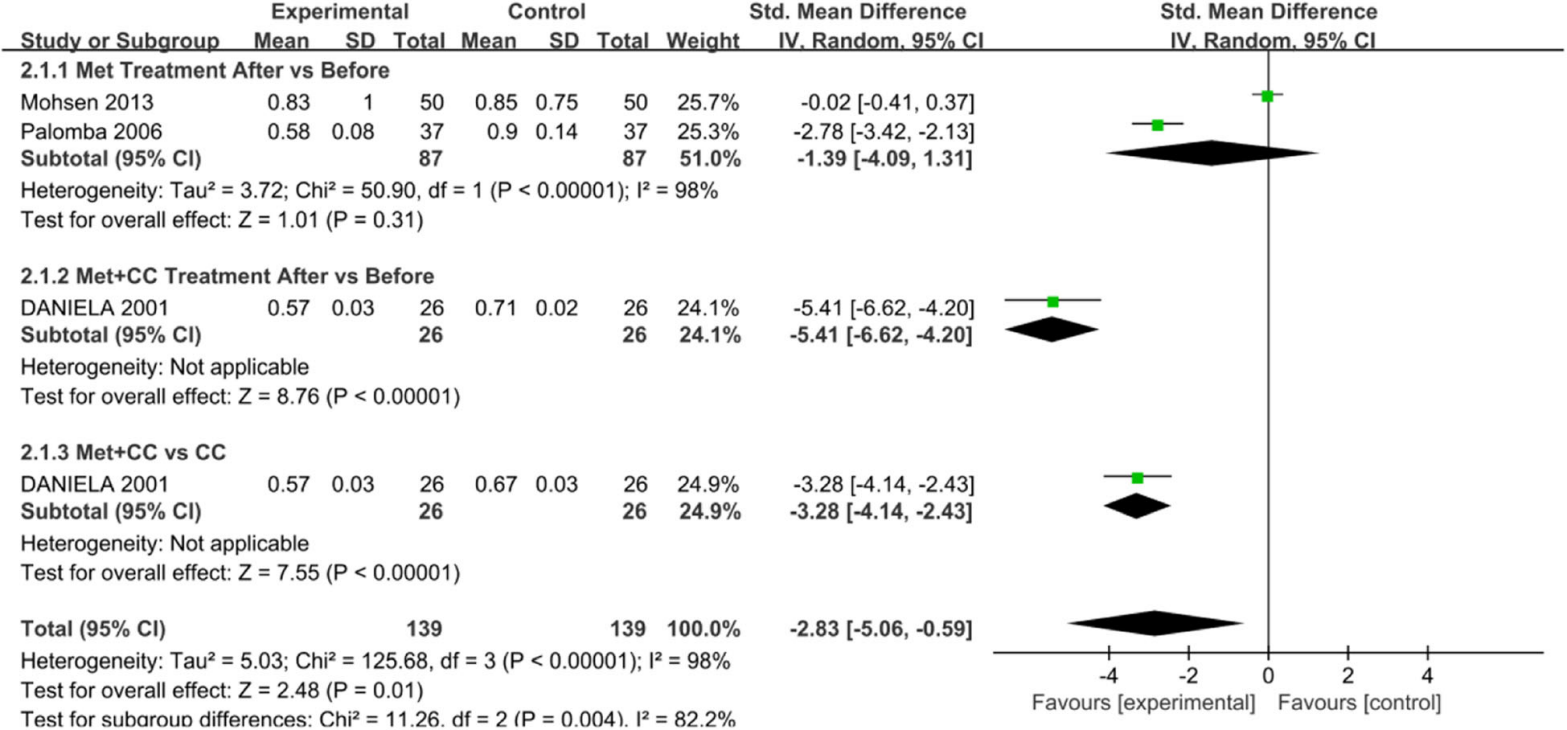
Forest plot of comparison: Met vs control, outcome: endometrium artery RI

**Fig. 5:**
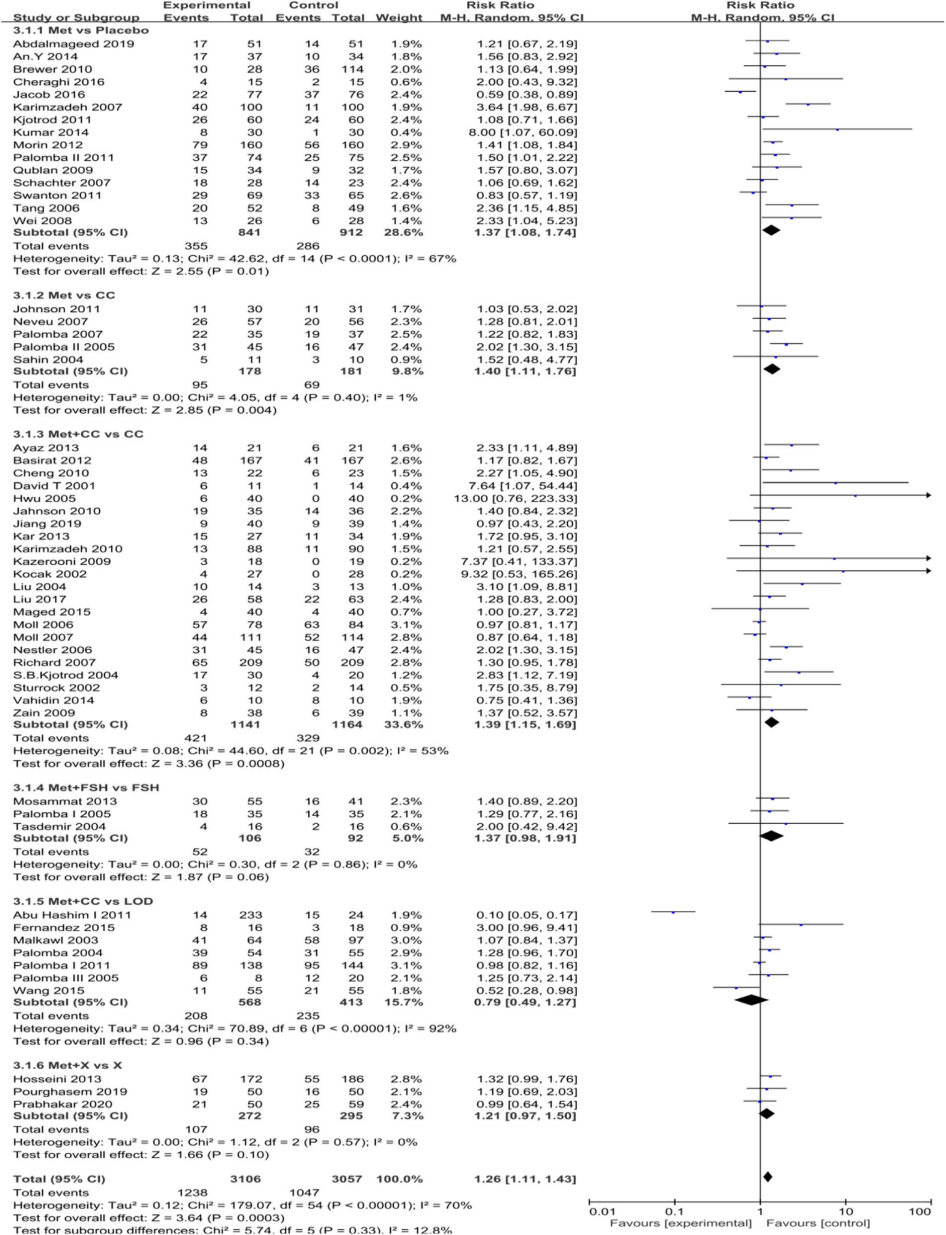
Forest plot of comparison: Met vs control, outcome: Clinical pregnancy rate (CPR)

**Fig. 6:**
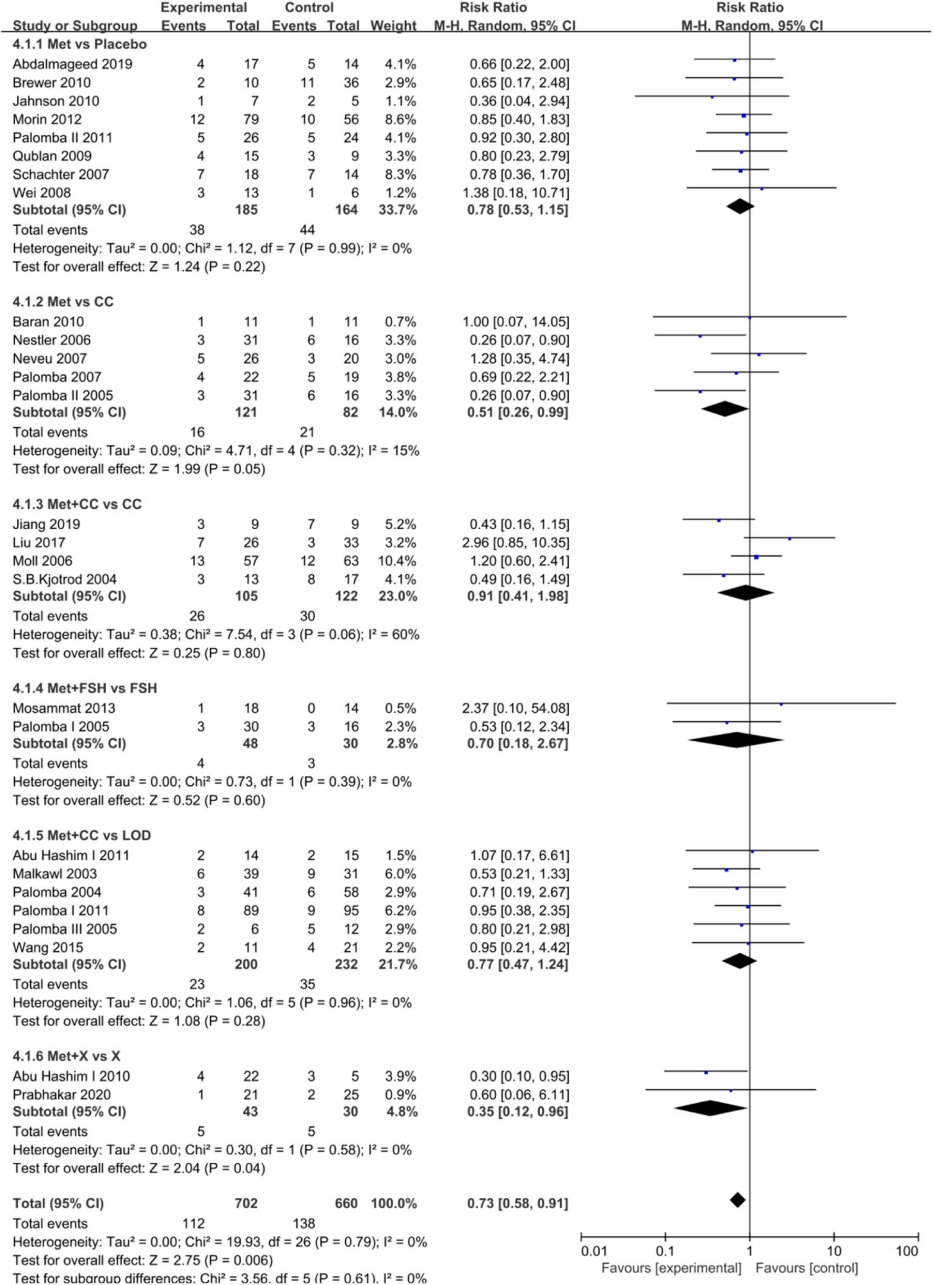
Forest plot of comparison: Met vs control, outcome: Miscarriage rate (MR)

Sixty-two eligible studies were included in the meta-analysis of metformin vs control. The intervention approach was slightly complicated between studies. Therefore, we analyzed the data in different subgroups. EMT and endometrium artery RI were considered-primary outcomes; the CPR and MR were secondary outcomes. Each outcome measurement is described in forest plots in Figs. 3, 4, 5 and 6.

#### Endometrial thickness (EMT)

EMT was determined in thirteen studies. The random-effects model showed that EMT in the metformin group was significantly thicker than that in the control group after treatment (SMD = 2.04, 95% CI (0.96,3.12), *P* = 0.0002). (Fig. 3).

#### Endometrium artery RI

The endometrium artery RI was reported in three studies. According to the random-effects model, the endometrium artery RI in the metformin group was significantly smaller than that in the control group after treatment (SMD = − 2.83, 95% CI: (− 5.06, − 0.59), *P* = 0.01). (Fig. 4).

#### Clinical pregnancy rate (CPR)

The CPR was described in Fifty-six studies. As revealed by the random-effects model, the clinical pregnancy rate in the metformin group was significantly different from that in the control group (RR = 1.26, 95% CI: 1.11–1.43, *P* = 0.0003). (Fig. 5).

#### Miscarriage rate (MR)

Twenty-seven studies included the MR. The random-effects model was applied, which indicated that the clinical pregnancy rate in the metformin group was significantly lower than that in the control group (RR = 0.73, 95% CI:0.58–0.91, *P* = 0.006). (Fig. 6).

### Publication bias analysis

**Fig. 7:**
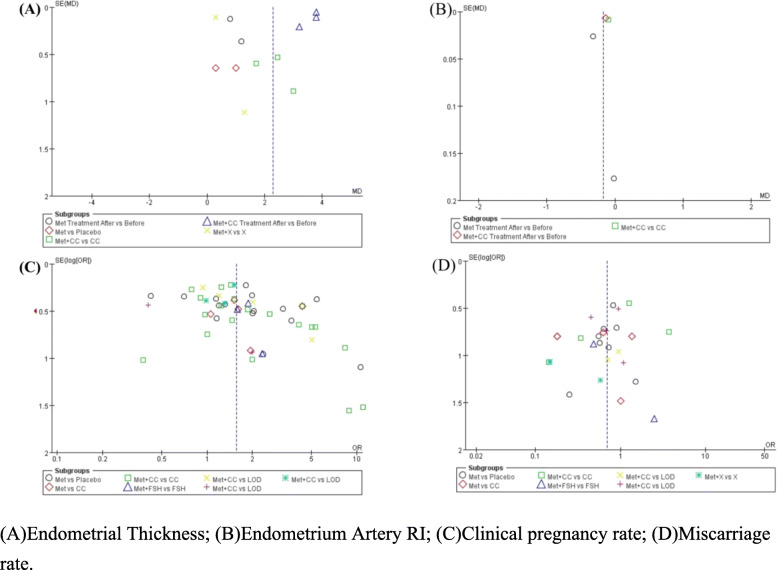
Funnel plot of comparison: Met vs control

In terms of publication bias estimation, as shown in Fig. 7, funnel plots indicated a relatively low likelihood of publication bias.

## Discussion

In recent years, endometrial factors have been responsible for 1/3 ~ 2/3 of pregnancies loss; however, the underlying mechanisms related to ER remain unclear and need further investigation [71]. It is well established that ER insufficiency is associated with several gynecological diseases, such as hydrosalpinx, endometriosis, and uterine fibroids [72]. In addition, PCOS, a pathological condition with high serum androgen levels, can impede ER. Some studies report several ER-related markers, including integrin, MMP-9, TMP1, VEGF and LIF, are significantly decreased in the endometrium of PCOS patients during the window of implantation compared with normal controls [73]. Other studies have found that HOXA-10 and IGFBP-1, molecules associated with embryo development and endometrial decidualization, are downregulated in the endometrium of PCOS patients. It is considered that the mechanism of ER reduction in PCOS patients may be explained in three aspects. First, PCOS patients usually experience amenorrhea or oligomenorrhea, which causes a relatively low progesterone level. Second, the progesterone-lacking endometrium undergoes long-term exposure to estrogen, which leads to a continuous proliferative phase of the endometrium and may eventually impede ER establishment. Third, PCOS patients usually have complications with insulin resistance and hyperinsulinemia. Moreover, glucose metabolism is important for endometrial preparation for embryonic implantation, especially for endometrial decidualization. The transport of glucose from outside to inside the cell is mediated by a kind of special transporter, GLUT, which is responsible for glucose intake in many tissues under the influence of insulin, including the human endometrium. Expression of GLUTs in PCOS patients with insulin resistance is significantly decreased in the endometrium compared with women with obesity but without PCOS. Glucose metabolism disorders may exacerbate ER and lead to a series of alterations in the endometrium, including ER molecular marker expression and macro indicators. Consistently, it has been reported that the serum level of insulin directly alters expression of ER-related markers.

Metformin, as a sensitizer of insulin, has been used for PCOS patients with insulin resistance to facilitate fertility restoration [74]. The application of metformin can increase GLUTs expression in the endometrium of PCOS patients; however, its effects possible mechanisms on ER are remain poorly understood.

In 2019, Jun Zhai reported that metformin could improve ER by downregulating expression of miR-491-3p and miR-1910-3p, thereby increasing expression of HOXA10 and ITGB3, known as important endometrial receptive markers, in the endometrium of PCOS women [75–77]. The expression level of these microRNAs also increased with up-regulating metformin concentration, showing a clear dose-dependent manner. Second, other studies have suggested that metformin inhibited estradiol and progesterone-induced endometrial stroma by regulating the p38 MAPK signaling pathway and by changing the expression of various cytokines, such as MMP-2, MMP-9, ER and PGR, which are involved in the decidualization of stromal cells [78]. Decidualization refers to the conversion from endometrial stromal fibroblasts to specialized secretory decidual cell, which provide a nutrient and immune privileged matrix and are essential for embryo implantation and placental development [79]. Third, some studies have documented that endometrial GLUT4 protein and mRNA expression were significantly increased after Met treatment in PCOS patients. Hyperglycemia and hyperinsulinemia may reduce GLUT4 expression in adipocytes, and Met can reverse this process and promote GLUT4 translocation [80]. GLUT4 express decreased in the endometrium may reduce glucose transmembrane transport, affect cell glucose utilization, and lead to endometrial embolism; subsequently, embryo implantation failure and miscarriage occur. However, it was also observed that expression of GLUT4 protein and mRNA in the endometrium of PCOS patients was increased after Met treatment. Therefore, Met may upregulate GLUT4 expression in the endometrium of PCOS patients and improve ER.

There is an assumption that Met can promote endometrium decidualization by increasing the expression of GLUT4 in the endometrium or providing enough energy for thickening of endometrium. Move over, it is also reported that it takes a long time to remodel and develop blood vessels in the placenta since the embryo implantation. At this stage, the embryo is exposed to a hypoxic environment. Changes in oxygen pressure cause multiple functional responses, such as adaptive responses to reduced oxygen concentrations or alternative metabolic pathways to provide oxygen energy substrates [81]. Therefore, we suggested that when the endometrial artery RI during embryo implantation is lower the blood supply for endometrium is better; that is, the RI of the endometrial artery is lower and more conducive to the remodeling and regeneration of blood vessels during embryo implantation. As mentioned above, treatment of PCOS with metformin can reduce the RI of the endometrial artery, and it is likely to provide a good environment for embryo implantation.

In this meta-analysis, the primary outcomes were EMT and endometrial artery RI. The main methods for evaluating ER are ultrasound, endometrial biopsy, endometrial fluid aspiration and hysteroscopy [82]. The application of ultrasound to evaluate ER can be divided into four aspects: EMT, endometrial volume, endometrial pattern and endometrial blood flow [83]. Currently, It is generally believed that EMT is the most common ER index. Some studies suggest a positive correlation between EMT and pregnancy rate [84–86], but there are also some reports no association [87]. Although the evaluation of ER in endometrial blood perfusion are constantly enriched, the most commonly used indicators are the PI and RI of the endometrial artery [88]. Many studies believe that the PI and RI of the endometrial artery can be used as effective indicators of ER [89, 90]. However, some suggest that measurement of the ER index of the uterine spiral artery or endometrial artery cannot reliably predict the prognosis of IVF [91–93].

This systemic review and meta-analysis summarize studies investigating the effect of metformin on ER of PCOS patients. It is still unclear how the protective effects of Met in endometrial receptivity operates, and further research is needed. Moreover, due to the high heterogeneity of the included data and the large merger bias, the results need to be treated with caution.

## Limitations

This meta-analysis had several limitations that should be taken into consideration when interpreting the conclusions. First, this meta-analysis summarized a total of 46 RCTs, 16 cohort studies, but the sample sizes were relatively small. Second, a poorly randomized design included studies, and the complex interventions made more biased in the study mergers. The results obtained were widely heterogeneous and considered to be the main limiting factor. According to the above limitations, caution should be used when evaluating the results of this meta-analysis.

## Implications

The following issues should be considered in future study design. First, as the time to measure EMT may have different effects on the study results, the time to measure EMT or ER in all patients should be the same period. Second, the current study involved few evaluation indicators for ER and Endometrium artery RI. Future research should add up more evaluation markers for ER.

## Conclusion

Overall, this systematic review and meta-analysis suggests that the effect of metformin for improving endometrial receptivity in women with PCOS is weak but meaningful. Notably, the sample size of the studies was not large, and the evidence was high quality albeit insufficient. Therefore, large-scale and multiple centers RCTs with rigorous methodological quality are needed to clarify the role of metformin in ER. Further research is needed to explore the long-term efficacy and the mechanisms of the intervention.

## Data Availability

All data are fully available without restriction.

## Abbreviations

Met: Metformin
PCOS: Polycyclic ovary syndrome
ER: Endometrial receptivity
EMT: Endometrial thickness
RI: Resistance index
CPR: Clinical pregnancy rate
MR: Miscarriage rate
PI: Pulsatility index
RCT: Randomized controlled trials
ARTs: Assisted reproductive technologies
IVF: In vitro fertilization
ET: Embryo transfer
